# The effects of different antihypertensive drugs on pain and joint space width of knee osteoarthritis – A comparative study with data from Osteoarthritis Initiative

**DOI:** 10.1111/jch.14362

**Published:** 2021-10-17

**Authors:** Mingyang Li, Yi Zeng, Yong Nie, Yuangang Wu, Yuan Liu, Limin Wu, Jiawen Xu, Bin Shen

**Affiliations:** ^1^ Department of Orthopedics, Orthopedic Research Institute, West China Hospital Sichuan University Chengdu Sichuan Province China

**Keywords:** antihypertensive drugs, hypertension, knee osteoarthritis

## Abstract

Hypertension was one common comorbidity of knee osteoarthritis (KOA), but the effect of different types of antihypertensive drugs on pain and joint space width (JSW) was unclear and not compared. Four hundred ninety KOA patients using one of the beta‐blockers, ACE inhibitors, angiotensin receptor blockers, Calcium channel blockers (CCBs), or thiazide diuretics were followed for four years. The blood pressure, cumulative knee replacement rate, Womac pain, and JSW were compared among groups. All data were from the Osteoarthritis Initiative project. The CCBs group has the highest systolic blood pressure, replacement rate, and pain score at most visit timepoints. At baseline, the CCBs group was with significantly higher pain score than the beta‐blockers group (3.3 vs 1.3, *p* < .05), the angiotensin receptor blockers group (3.3 vs 1.4, *p* < .05), and the thiazide diuretics group (3.3 vs 1.6, *p* < .05) in male; the CCBs group was with significantly higher pain score than the beta‐blockers group (3.8 vs 2.0, *p* < .01), and the angiotensin receptor blockers group (3.8 vs 2.2, *p* < .05) in female. The results of females at 36 months were similar to the baseline. Among the common antihypertensive drugs, CCBs were associated with high replacement rates, high pain scores, and less JSW in KOA patients.

## 1 INTRODUCTION

Knee osteoarthritis (KOA) is a significant health problem with a substantial health care burden associated with pain, reduced joint function, diminished quality of life. With the global increase of aging and obesity, this burdensome issue is becoming more prevalent.^1^ Osteoarthritis is a complex chronic disease, frequently accompanied by the presence of multimorbidity.

Hypertension and OA are both highly prevalent diseases, and hypertension has been found related to knee OA in previous studies.^2^, ^3^, ^4^, ^5^ Due to the shared risk factors, such as aging, obesity, and chronic inflammation,^6^, ^7^ hypertension, and KOA were common comorbidities. Moreover, multiple biomolecular components are involved in both hypertension and knee OA. For example, proinflammatory cytokine interleukin‐6 acts in both hypertension and knee OA.^8^, ^9^ Metabolic factors might also play a role in the comorbidity of hypertension and KOA. Metabolic syndrome‐related OA was classified as a particular phenotype in some epidemiological studies.^10^, ^11^

Antihypertensive drugs were routinely applied in KOA patients accompanied by hypertension. However, the optimal choice for them was still unclear. Valdes and colleagues reported that the use of beta‐blockers is associated with less joint pain and opioids consumption in individuals with symptomatic OA,^12^ while Zhou and colleagues detected no evidence of beta‐blocker use in reducing knee pain or analgesic drug use.^13^ Wu and colleagues demonstrated that the local renin‐angiotensin system is important for regulating the homeostasis of the chondrocyte, and ACE inhibitors or angiotensin receptor blockers might be potential in KOA therapy.^14^ Calcium channel blockers (CCBs) might also affect KOA since calcium channels are essential for neuronal excitability and play a role in pain genesis. Driban and colleagues reported evidence of thiazide diuretics on KOA symptom modification.^15^ In brief, those five types of common antihypertensive drugs: beta‐blockers, ACE inhibitors, angiotensin receptor blockers, CCBs, and thiazide diuretics, might exert different effects on KOA. However, no direct comparison of KOA pain and structural progression concerning each type of antihypertensive drug was conducted.

This study compares the pain and radiologic severity of KOA patients who use different types of antihypertensive drugs and the subsequent changes in 4‐year follow‐up.

## 2 METHODS

In the Osteoarthritis Initiative project (OAI, https://nda.nih.gov/oai), persons aged from 45 to 79 years with risk factors for KOA or symptomatic KOA were prospectively recruited and followed. Subjects with inflammatory arthritis, with severe joint space narrowing in both knees, with unilateral knee joint replacement and severe joint space narrowing in the contralateral knee, with walking aids for the most time were excluded. OAI project obtained approval from the institutional review boards at corresponding sites, and all the participants signed informed consent.

**FIGURE 1:**
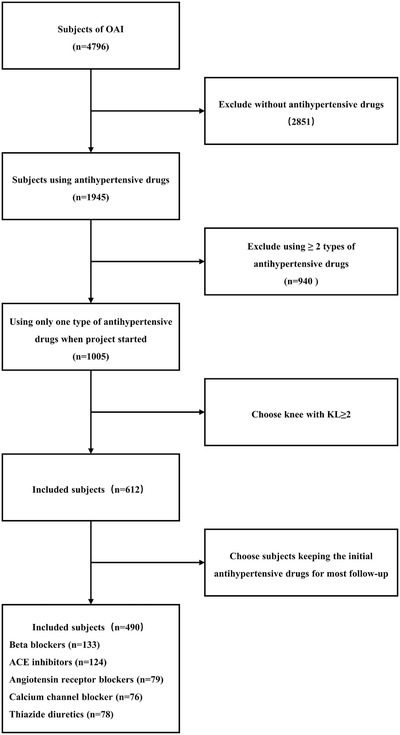
The flowchart of selecting the eligible subjects

Among the 4796 particpants in the OAI project, 1945 patients used antihypertensive drugs. To eliminate the confounding effect from the interaction between antihypertensive drugs, only 1005 patients with a single type of antihypertensive drug regimen were included in the analysis. Then, only knees with Kellgren Lawrence (KL) grade ≥2 were included. When both knees of one subject were qualified, only one knee was selected out randomly. Patients who changed the initial antihypertensive drugs in more than half the follow‐up time were excluded. Finally, 133 patients using beta‐blockers, 124 patients using ACE inhibitors, 79 patients using angiotensin receptor blockers, 76 patients using CCBs, and 78 patients using thiazide diuretics were included in this study (Figure 1).

To identify the medication inventory, participants were asked to bring all prescription medications that they took during the last 30 days to the researchers at the visit. If they did not bring all prescription medications, a telephone call to the participant 1–2 days after the visit would be performed to complete medication data collection. The antihypertensive drugs investigated in this study were classified and presented in Table 1.

**TABLE 1:** Detailed information on the antihypertensive drugs

Beta blockers	N	ACEi	N	ARB	N	CCB	N	Diuretics‐thiazide	N
Acebutolol	2	Benazepril	3	Candesartan	5	Amlodipine	31	Hydrochlorothiazide	78
Atenolol	67	Captopril	2	Irbesartan	12	Diltiazem	14		
Betaxolol	1	Enalapril	15	Losartan	18	Felodipine	5		
Bisoprolol	2	Fosinopril	2	Olmesartan	10	Nifedipine	11		
Carteolol	2	Lisinopril	81	Telmisartan	2	Nisoldipine	1		
Levobunolol	2	Quinapril	14	Valsartan	32	Verapamil	14		
Metoprolol	31	Ramipril	5						
Nadolol	4	Trandolapril	2						
Propranolol	9								
Sotalol	2								
Timolol	11								

Baseline characteristics were extracted and compared, including age, sex, body mass index (BMI), Charlson comorbidity index, KL grade, and antihypertensive drug use frequency. For joint space width (JSW) measurement, standard posteroanterior fixed‐flexion knee radiographs were acquired at baseline, 12‐month, 24‐month, 36‐month, and 48‐month. Quantitative measurements of tibiofemoral JSW are made from digitized knee images by automatic software.^16^ Knee pain was evaluated by Western Ontario and McMaster Universities Osteoarthritis Index (Womac) pain subscale (score range 0–20).

Kaplan‐Meier (KM) curve^17^ was applied to show the survival rate with knee replacement surgery as the endpoint. The Log‐Rank test was used to detect the difference in survival rate between groups. One‐way ANOVA was applied to investigate the difference of continuous variables among the groups. The Student's *t*‐test was adopted to compare the mean of two different groups. The difference of categorical variables was tested by the chi‐square test. A two‐tailed *p*‐value of < .05 was considered significant. Statistical analysis was conducted with SPSS software (version 25.0; SPSS Science, Chicago, Illinois, USA).

## 3 RESULTS

Among the five types of antihypertensive drug groups, the age distribution, mean BMI, mean CCI, KL grade distribution, and antihypertensive use frequency showed no significant difference. However, the percent of females varied from 50% in the ACE inhibitors group to 74% in the CCBs group (Table 2). Since the sex distribution was significantly different, the following analysis was stratified by sex to reduce the bias.

**TABLE 2:** Basic characteristics of each group

	Beta blockers	ACE inhibitors	Angiotensin receptor blockers	Calcium channel blocker	Thiazide diuretics	*p* value
Number	133	124	79	76	78	–
Age (< 50/50‐60/60‐70/≥70)	4%/16%/36%/44%	5%/31%/36%/27%	5%/24%/46%/25%	3%/25%/37%/36%	5%/17%/41%/37%	.10
Sex (male percentage)	36%	50%	39%	26%	27%	.003
BMI	28.99± 4.57	30.37 ± 4.82	30.18± 4.93	30.44 ±4.06	29.63 ± 4.85	.10
Charlson comorbidity score	0.42 ± 0.83	0.48 ± 0.97	0.54 ± 1.04	0.42 ± 0.79	0.33 ± 0.72	.62
KL grade (2/3/4)	53%/38%/5%	58%/30%/10 %	57%/35%/8%	57%/33%/7%	58%/32%/9%	.85
Drug use frequency (regular/as need)	123/10	110/14	72/7	68/8	62/15	.10

### 3.1 Blood pressure

**FIGURE 2:**
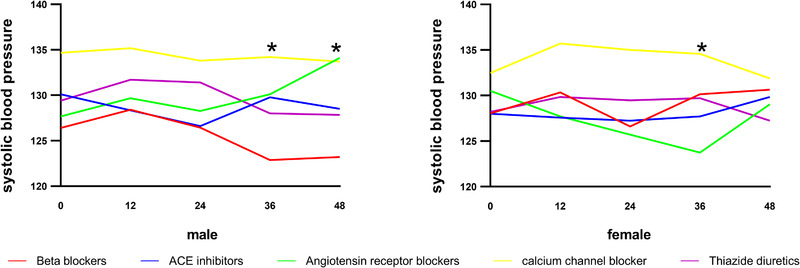
The comparison of blood pressure in the five groups in 4‐year follow‐up

At most timepoints, the mean systolic blood pressure in the CCBs group was highest (Figure 2). In males, the value of the CCBs group was significantly higher than the beta‐blockers group at 36‐month (134.2 vs 122.9, *p* < .05) and 48‐month (133.7 vs 123.2, *p* < .05). In females, the value of the CCBs group was significantly higher than the angiotensin receptor blockers group (134.6 vs 123.8, *p* < .05) at 36 months.

### 3.2 Replacement

**FIGURE 3:**
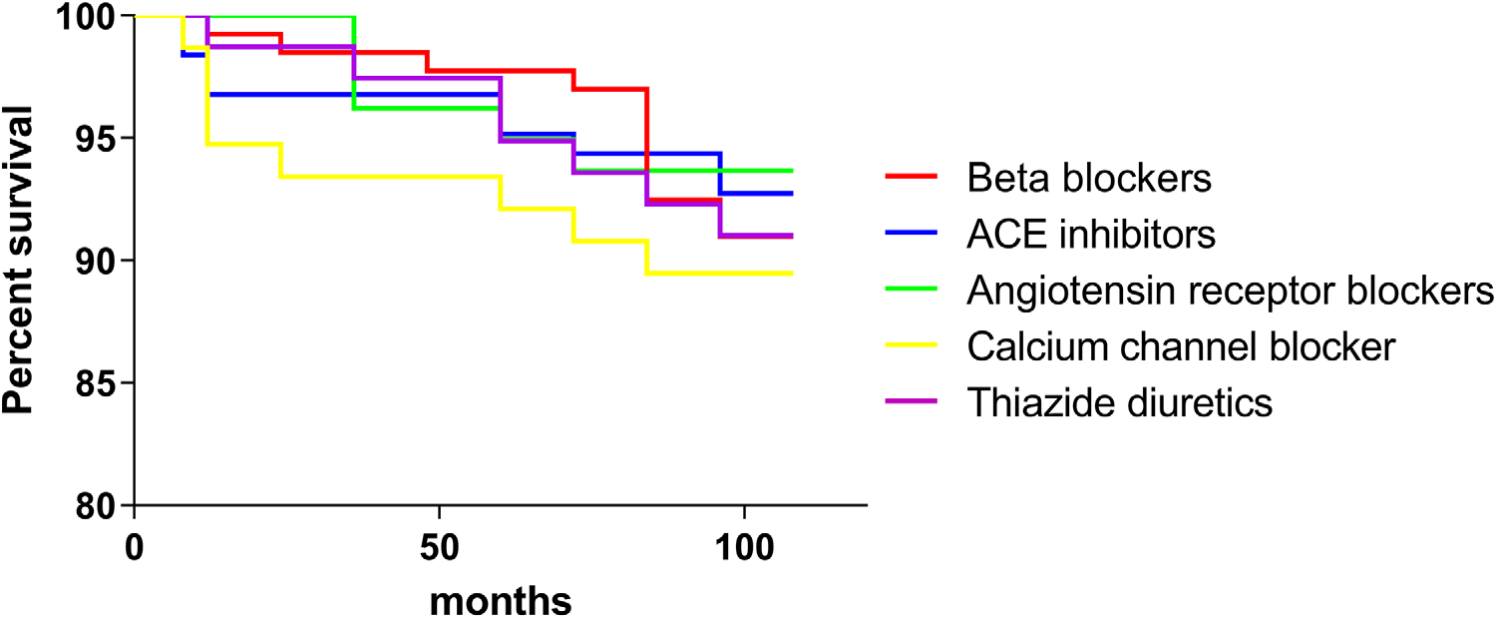
The survival curve of the five groups in 9‐year follow‐up, with knee replacement as the endpoint

In the KM curve, the CCBs group seemed to have a higher rate of knee replacement in a 9‐year follow‐up (Figure 3). However, the Log‐Rank test presented a *p* value of .77, suggesting the difference between the groups was not statistically significant. Due to the overall replacement number was small, stratified analysis by sex was not conducted.

### 3.3 Pain

**FIGURE 4:**
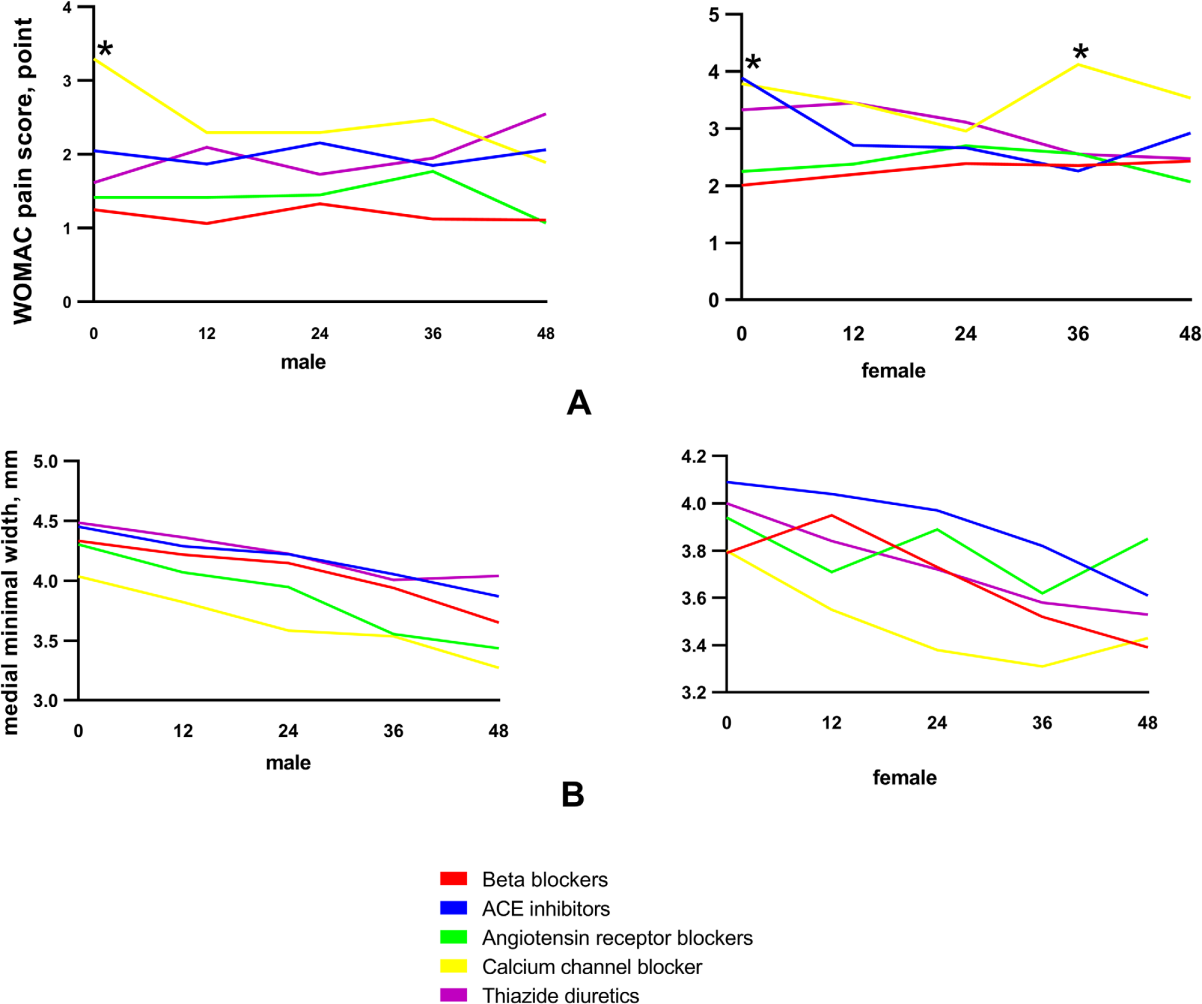
The comparison of Womac pain (A) and medial minimal JSW (B) in the five groups in 4‐year follow‐up

In the 4‐year follow‐up, the CCBs group had the highest mean Womac pain score at most visit time points (Figure 4A). In males, the mean Womac pain score in the CCBs group was significantly higher than the beta‐blockers group (3.3 vs 1.3, *p* < .05), the angiotensin receptor blockers group (3.3 vs 1.4, *p* < .05), and the thiazide diuretics group (3.3 vs 1.6, *p* < .05) at baseline. In females, the mean pain score in the CCBs group was significantly higher than the beta‐blockers group (3.8 vs 2.0, *p* < .05), and the angiotensin receptor blockers group (3.8 vs 2.2, *p* < .05) at baseline; at 36‐month, the mean pain score in the CCBs group was significantly higher than any of the other group (CCBs = 4.1, beta‐blockers = 2.4, ACE inhibitors = 2.3, angiotensin receptor blockers = 2.6, thiazide diuretics = 2.6).

### 3.4 JSW

In both males and females, the CCBs group had the narrowest medial minimal JSW in the five groups at the most visit period (Figure 4B). However, there was no significant difference among the five groups no matter in males or females.

In the CCBs group, patients were divided into dihydropyridine and non‐dihydropyridine. No significant difference was detected between the two subgroups in systolic blood pressure, pain, and joint width at any time points (Supplementary 1).

## 4 DISCUSSION

The effect of different types of antihypertensive drugs on KOA pain and structural change was unclear and not compared. We analyzed the blood pressure, knee replacement rate, Womac pain, and JSW in patients with beta‐blockers, ACE inhibitors, angiotensin receptor blockers, CCBs, and thiazide diuretics. CCBs were associated with higher joint replacement rate, more pain, and narrower JSW in KOA patients in the 4‐year follow‐up.

The blood pressure control results were quite unexpected because previous studies demonstrated that the interaction between NSAIDs and CCBs was less than the interaction between NSAIDs and other antihypertensive drugs. The interaction between NSAIDs and antihypertensive drugs mainly depends on the role that prostaglandins play in the antihypertensive mechanism,^18^, ^19^ while the antihypertensive effect of CCBs is not dependent on the action of prostaglandins.^20^ Theoretically, CCBs should be less affected by NSAIDs, and previous researches also supported this.^21^, ^22^, ^23^ However, our results were the opposite. The potential explanations lied in two aspects. First, the effect of NSAIDs on the antihypertensive drugs varied with the type of NSAIDs.^18^, ^20^, ^21^, ^22^, ^23^, ^24^, ^25^ Considering that KOA patients might use variable NSAIDs regimens in the follow‐up period, the heterogeneity from NSAIDs was hard to eliminate. Second, the dosage of CCBs might be suboptimal due to the concern about the upper gastrointestinal bleeding.^26^, ^27^, ^28^ We could not verify this assumption because the OAI database did not collect the data about the dosage. Further prospective researches were necessary to clarify the antihypertensive effect of CCBs in osteoarthritis patients.

It was not investigated whether CCBs caused the progression of KOA in previous studies. Although arthralgia after using the CCBs has been reported,^29^, ^30^ the mechanism was still unknown. It was inferred that the CCBs affect KOA via multiple potential pathways. First, CCBs might directly influence the chondrocyte activities. Kaplan and colleagues demonstrated that Nimodipine impaired chondrocyte proliferation and damaged extracellular matrix structures in vitro.^31^ Second, the vasodilating effects of CCBs might be impaired by NSAIDs. Pavlicević and colleagues found that NSAIDs did not significantly change body weight, urinary output, serum creatinine, or serum/urinary electrolyte profile, and they concluded that NSAID's pro‐hypertensive effects seem mainly due to vasoconstriction rather than volume expansion.^32^ The antagonistic action on the vessel between CCBs and NSAIDs might lead to poorly controlled hypertension, and therefore KOA patients using CCBs experienced more progression. Third, skeletal muscle might be the mediator between CCBs and KOA progression. Although skeletal muscle does not depend on extracellular calcium for muscle contraction, the CCBs also exert pharmacological effects on the skeletal muscle, such as glucose transport.^33^ Considering that skeletal muscle may play an essential role in OA pathogenesis through its function, tissue, and molecular mechanisms,^34^ researches about skeletal muscle and CCBs in KOA patients were necessary.

In our results, beta‐blockers have favorable performance in KOA patients with relatively low pain scores. The beta‐adrenergic receptor (b‐AR) system has been recently targeted in pain management and drug development.^35^, ^36^ Specific beta‐adrenergic receptors are located on peripheral nociceptors, superficial dorsal horn, and dorsal root ganglia, resulting in hyperalgesia.^37^ Martin et al. reported that bupranolol, a beta‐blocker, had good antinociceptive efficacy and safety in mouse models of chronic pain.^35^ Valdes and colleagues supported that beta‐blockers were associated with lower Womac pain scores and with a lower risk of joint pain (OR = 0.68, 95%CI = [0.51, 0.92]; *p* < .01), with non‐beta‐blocker users as a control.^12^ Nakafero and colleagues also reported a reduced cumulative risk of knee pain in the beta‐blockers users than non‐users.^38^ Our results revealed the benefit of beta‐blockers in pain management of KOA, and more importantly, the main difference between this study and previous studies was that we directly compared different types of antihypertensive drugs. Zhou and associates took all of the non‐beta‐blockers antihypertensive drugs as a control, and they detected no evidence of beta‐blockers use in reducing knee pain.^13^ This study classified antihypertensive drugs into five types and found significant difference between beta‐blocker and CCBs.

However, there are several limitations. First, the OAI project did not collect the dosage of antihypertensive drugs, and different dosages might cause potential bias in this study. Futural high‐quality RCTs were required to verify our results. Second, one type of drug was integrated as one group in the analysis, which might cause some heterogeneity. For example, β1/β2 adrenoreceptor selectivity was not specially considered in the beta‐blockers group. Although grouping patients with a single drug would be more convincing, it was difficult to collect enough eligible participants. Third, we could not clarify the causal relationship between the higher pain and higher blood pressure in the CCBs group. Further researches were still necessary to reveal the original trigger factor. Fourth, although we used prospective data of OAI, no randomization was applied for our study purpose, and confounding factors might still exist. Fifth, the sample size of this study was not large. Nation‐wide database analysis in the future might provide extra solid evidence.

## 5 CONCLUSION

In the KOA patients using common antihypertensive drugs, high replacement rate, high pain score, and less JSW were observed in subjects with CCBs. More high quality RCTs are necessary to clarify the exact effect of CCBs in KOA.

This study was funded by National Natural Science Foundation of China (Program No. 81974347), National Clinical Research Center for Geriatrics, West China Hospital, Sichuan University (NO. Z20192003), and Science&Technology of Foundation of Sichuan province of China (2021YFH0094). All authors declared that the funding did not has any effect on the results of this study.

## AUTHOR CONTRIBUTIONS

Study design and manuscript writing (LMY, NY, SB); Data extracting (WYG, ZY) Statistical analysis (WLM, LY, XJW); Data checking (LMY, SB).

## ETHICAL APPROVAL STATEMENT

All procedures performed in studies involving human participants were in accordance with the ethical standards declaration and its later amendments or comparable ethical standards. OAI project was approved by the institutional review boards at different sites.

## CONFLICT OF INTEREST

The authors have declared that no competing interests exist.

## Supporting information

Supporting Information
