# Supplementary figures and images for: The effects of different antihypertensive drugs on pain and joint space width of knee osteoarthritis – A comparative study with data from Osteoarthritis Initiative
Source: J Clin Hypertens (Greenwich). 2021 Oct 17;23(11):2009–15. doi: 10.1111/jch.14362 (PMC8630607; doi:10.1111/jch.14362)

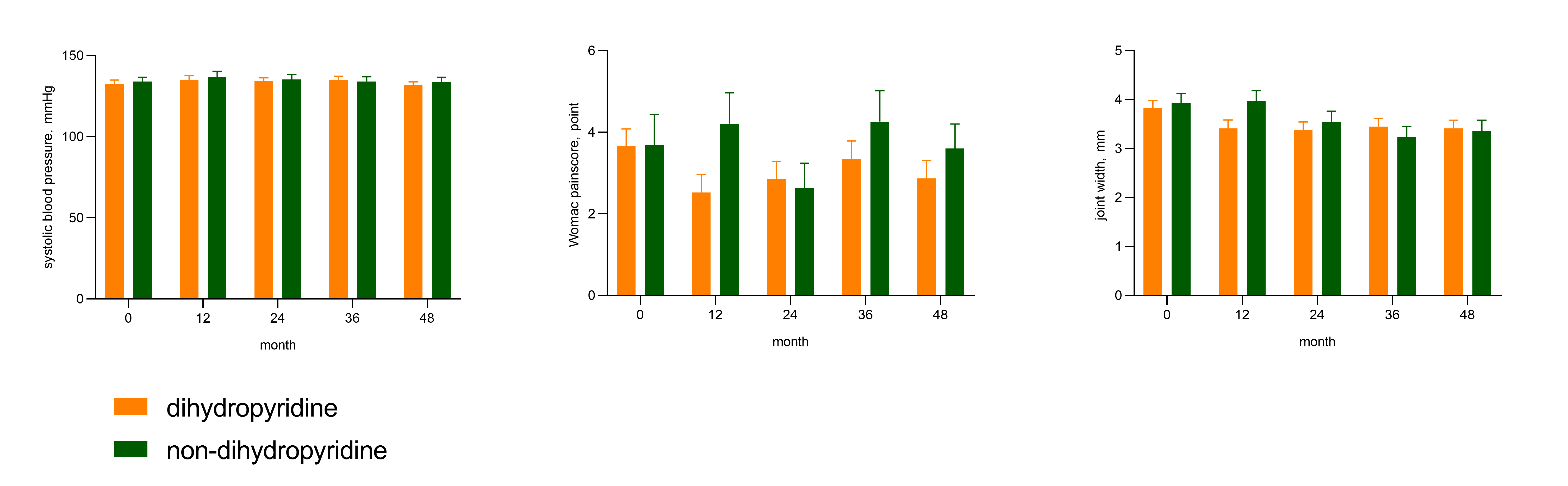

Supplement: Supplementary file 1 — Supporting Information [file JCH-23-2009-s001.tif]
